# Attitudes of Vietnamese University students on restrictions of rights and compulsory admissions in patients with severe mental illness – a cross-sectional study

**DOI:** 10.3389/fpsyt.2025.1542247

**Published:** 2025-03-19

**Authors:** Solveig Kemna, Van Tuan Nguyen, Kerem Böge, Malek Bajbouj, Max Bringmann, Sebastian Weyn-Banningh, Luisa Eilinghoff, Van Phi Nguyen, Laura Elisabeth Tuturea, Thien Le Cong, Thi Thu Ha Le, Thi Minh Tam Ta, Eric Hahn

**Affiliations:** ^1^ Department of Psychiatry and Neuroscience, Charité University Medicine Berlin, Berlin, Germany; ^2^ Department of Psychiatry, Hanoi Medical University, Hanoi, Vietnam; ^3^ National Institute of Mental Health, Bach Mai Hospital, Hanoi, Vietnam; ^4^ Department of Mental Health, National Geriatric Hospital, Hanoi, Vietnam

**Keywords:** medical students, psychiatry, stigma, restrictions, compulsory admissions, mental health literacy

## Abstract

**Introduction:**

This cross-sectional, explorative study examines university students’ attitudes (n = 610) in Hanoi, Vietnam, toward the rights of psychiatric patients.

**Methods:**

Medical students responded to self-report questionnaires investigating their attitudes towards restrictions and compulsory admissions in case of severe mental illness after attending a psychiatry course. Medical students and non-medical students who did not participate in the course served as two control groups.

**Results:**

In all groups, the majority of students opposed restricting the civil rights of psychiatric patients, but most supported compulsory admissions in certain situations. Medical students who had not attended a psychiatry course were generally more in favor of compulsory admissions compared to those who had attended a psychiatry course and non-medical students. However, when investigating attitudes on compulsory admission in specific scenarios, students that had attended a psychiatry course were more likely to endorse compulsory admissions, except when admission was based on the patient’s family request.

**Discussion:**

Medical and psychiatric training seem to encourage more differentiated opinions on the use of compulsory admissions in psychiatric care. Future research, including longitudinal designs and a broader geographical scope, is needed to better understand the impact of psychiatric education in medical studies on attitudes toward mental health.

## 1 Introduction

Although mental health care was defined as a national priority by the government in Vietnam, psychiatric infrastructure remains insufficient (1, 2). For every 100.000 inhabitants, there are 0.62 psychiatrists, and only 143 clinical psychologists and psychotherapists in the public health sector overall (3, 4). Additionally, the general population and many health professionals show limited mental health literacy (5, 6). This has been attributed at least partly to misinformation in the media regarding mental health, as well as the low number of licensed non-medical mental health professionals and psychiatrists (4). Negative attitudes toward psychiatry as a medical profession may discourage medical students from following a career in this field (7, 8). Moreover, undergraduate medical students often have only limited knowledge of psychiatric diagnoses and treatment, such as depression and anxiety, and show low proficiency in providing first-aid support to these populations (9–12).

This lack of understanding of mental illnesses and knowledge of psychiatric care can lead to negative beliefs and social distancing from psychiatric patients (13, 14) and, in turn, negatively impact the quality of provided health care and help-seeking behavior (10, 15, 16). Furthermore, societal stigmatization of psychiatric patients can also have broader implications. In many countries, persons with mental health disorders are still faced with the restriction of civil rights such as voting (17–19).

Attitudes toward the mental health care system and psychiatric conditions are also reflected in views on the role of compulsory admissions, i.e., admitting psychiatric patients – under certain conditions – against their will to a hospital. On the one hand, advocating for stricter policies of compulsory admissions can be interpreted as a sign of a desire for social distance and negative attitudes towards psychiatric patients as they might be deemed dangerous. On the other hand, support for compulsory admissions can also be a signal of trust in the mental health care system as a place where patients receive appropriate support. A differentiated assessment of admission conditions provides insights into population’s attitudes toward mental health care (20). Previous studies in an urban Vietnamese population have shown a preference for caring for psychiatric patients within families instead of admitting them to a hospital (8).

Although globally, attitudes towards the restrictions of rights and compulsory admission of psychiatric patients have been researched (20–31), in Vietnam specifically research is scarce and has focused on attitudes toward restrictions and compulsory admissions in the general population (32).

Overall, mental health stigma can be reduced by improving knowledge and enabling contact with persons with mental disorders (33). In medical professionals specifically, mental health literacy can be improved by including mandatory psychiatry courses in the medical curriculum (13, 14, 34–47). This has previously been demonstrated in Vietnamese medical students, who hold more benevolent attitudes towards psychiatric patients than non-medical students, and a psychiatry course positively impacted opinions on psychiatry (9). However, research on the attitudes of Vietnamese students toward psychiatric patients and mental health literacy remains scarce.

This cross-sectional, explorative study aimed to assess the attitudes of Vietnamese students towards the restriction of civil rights and compulsory admissions of patients with severe mental illness. Specifically, attitudes of medical students that had attended a psychiatry course, those that had not yet attended one, and non-medical students were compared. Generally, it was hypothesized that higher mental health literacy is associated with lower endorsement of civil rights infringements of psychiatric patients. Overall, this study aims to contribute to research on non-Western attitudes towards severe mental illnesses.

## 2 Methods

### 2.1 Study participants

Data from university students in Hanoi, Vietnam, was collected between September 2020 and May 2021. After excluding 15 participants due to incomplete data, the total sample (n = 610) comprised medical students from Hanoi Medical University (HMU), including those who had already attended a psychiatry course (MSPS n = 168), and two control groups: medical students who had not yet taken the course (MSNPS; n = 223) and non-medical students (NMS; n = 219) from the Hanoi University of Science and Technology (HUST). Ethical approval was obtained from the ethical committee of HMU (IRB-VN01.00IIIRB00003121IFWA00004148). All participants provided informed consent before data collection.

### 2.2 Psychiatry course

The psychiatry course was part of the regular curriculum of the fifth semester of medical school at HMU. The course duration was four weeks and consisted of two parts. Firstly, the medical students attended a one-week theoretical course on psychiatric diseases, diagnostic procedures, and treatment options. Secondly, students interned at a general psychiatric hospital for three weeks. Here, they assisted physicians and participated in individual therapy sessions. The course ended after passing a final exam (9).

### 2.3 Self-report questionnaires

MSPS filled out self-report questionnaires after participating in a psychiatry course. MSNPS filled out self-report questionnaires before participating in a psychiatry course. NMS received questionnaires at a non-specific point during the study period. One aim of the study was to investigate the impact of psychiatric training on students’ general perceptions and attitudes. Therefore, no explanations or descriptions about the nature of severe psychiatric disorders was provided prior to the interviews. All items included in the final questionnaires were jointly decided on by the Global Mental Health research team of the Department of Psychiatry, Charité - Universitätsmedizin Berlin and researchers and psychiatrists from the Department of Psychiatry, HMU and the associated National Institute of Mental Health (NIMH). The translation and back-translation methods were used to provide questionnaires in Vietnamese (48).

First, socio-demographic data, including gender, age, and marital status, were assessed. Additionally, information on choosing a medical discipline as a profession, previous contact with the psychiatric healthcare system, having a family member diagnosed with a psychiatric illness, and religious beliefs was obtained. Furthermore, participants received eleven questionnaires assessing attitudes toward stigmatization and discrimination of mental illness and mental health care in general. Attitudes towards restrictions and compulsory admissions in psychiatric patients were assessed by a questionnaire previously used in a study by 22, which assessed agreement with restricting certain individual rights of patients with severe psychiatric disorders (22). These included the right to vote, possession of a driver’s license, being in favor of abortion if a mentally ill female becomes pregnant, and whether sterilization in persons with a psychiatric illness is favored. To assess attitudes toward compulsory admissions in case of severe mental illness, respondents were asked whether a mentally ill person should, under certain conditions, be admitted to a hospital, even if it is against their will. Subsequently, different specific scenarios are described in which a patient could be admitted against their will, such as suicidality, violence towards others, self-neglect, delusions, social withdrawal, non-adherence to a prescribed medicine, causing a public disturbance, and upon the request of the patient’s family (22).

### 2.4 Statistical analysis

Statistical analyses were calculated with R (Version 4.3.1, 2023). First, socio-demographic data, including gender (binarized into female and male), age (in years), and marital status (binarized into “no partner” and “in partnership”), living area (binarized into “city and larger” and “< city”), semester (binarized into “At least fourth academic year” and “< fourth academic year”), considering oneself mentally ill, and having a close person who had been to a mental health practitioner were assessed. Socio-demographic variables were described by frequency, percentage, median, and interquartile range (**Table 1**). The first question was excluded from the analysis of attitudes towards restrictions as it assessed attitudes on compulsory admissions, which was already included in the compulsory admissions questionnaire. Additionally, the last question - assessing agreement to sterilization of mentally ill people against their will - was excluded since the previous question already assessed agreement to sterilization in general. Next, a sum score of the responses across the remaining four questions on restrictions was created for the primary analysis. Results were binarized into “at least one question answered ‘Yes’” and “no question answered ‘Yes’”. Then, two logistic regression analyses were calculated to capture all three comparisons between the student groups, with the first model employing MSPS, and the second model NMS as reference category. For both models, all demographic variables included in analyses were used as covariates except for age, since there was little variation in age across the sample. In a secondary analysis, group comparisons were calculated separately for each item of the Restrictions questionnaire using *χ²-*tests. This two-step analysis was selected to have an overall comparison between attitudes while considering covariates without losing statistical power due to correction for multiple comparisons. The secondary analysis aimed to generate tentative evidence on more nuanced attitudes in the study population.

**Table 1:** Demographics of study sample.

Variable	Overall, N = 610^a^	MSPS, N = 168^a^	MSNPS, N = 223^a^	NMS, N = 219^a^
Gender				
Male	309 (51%)	72 (43%)	117 (52%)	120 (55%)
Female	301 (49%)	96 (57%)	106 (48%)	99 (45%)
Age	22.00 (20.00, 22.00)	22.00 (22.00, 23.00)	22.00 (22.00, 22.00)	19.00 (19.00, 20.00)
Living area				
< city	300 (49%)	63 (38%)	137 (61%)	100 (46%)
City	310 (51%)	105 (63%)	86 (39%)	119 (54%)
Relationship status				
In partnership	195 (32%)	70 (42%)	73 (33%)	52 (24%)
No partner	415 (68%)	98 (58%)	150 (67%)	167 (76%)
Semester				
< 4th year second year	171 (28%)	0 (0%)	0 (0%)	171 (78%)
At least fourth year	439 (72%)	168 (100%)	223 (100%)	48 (22%)
Considers oneself mentally ill	92 (15%)	40 (24%)	28 (13%)	24 (11%)
Close person has been to mental health practitioner	167 (27%)	40 (24%)	66 (30%)	61 (28%)

a n (%); Median (IQR).

Similarly, for the general analysis of the attitudes toward compulsory admissions, two logistic regressions were calculated for the first question on compulsory admissions to compare the general attitudes of all three student groups. The same covariates as in the previous models were used. Next, as a secondary analysis, group comparisons for each subsequent item describing a specific scenario were calculated separately with *χ²-t*ests. The level of significance was set at p <0.05.

## 3 Results

The analyzed sample showed a balanced gender distribution (301 females; 309 males). 168 (27.2%) participants had attended a psychiatry course, 223 (36.1%) were medical students who hadn’t attended a psychiatry course, and 219 (35.5%) participants were students at the HUST. It can be noted that 11 to 24% of students considered themselves to be mentally ill. This question was aimed at understanding the respondent’s self-assessment of the likelihood that they were mentally ill, not whether a specific mental disorder had been professionally diagnosed. The participants were not asked to disclose the nature of a potentially diagnosed mental illness. An overview of the distribution of socio-demographic data can be found in **Table 1**. The three groups show significant differences in the gender distribution, with more male students in the NMS than in the MSPS (*χ²* = 4.95; p = 0.026). Furthermore, MSPS (*χ²* = 13.33; p < 0.001) and MSNPS (*χ²* = 3.97; p = 0.046) were significantly more often in a relationship than NMS. Group comparisons can be found in the appendix.

Across all groups, the majority of the study sample did not support restricting the rights of persons with mental illness. However, 55.7% of respondents endorsed restricting psychiatric patients’ right to vote. 5.2% of participants reported being in favor of revoking the driver’s license of someone who has been treated once in a mental hospital. Overall, 7.5% of participants were in favor of abortion if a mentally ill female becomes pregnant, and 15.4% agreed with sterilizing persons with severe mental illness.

**Figure 1:**
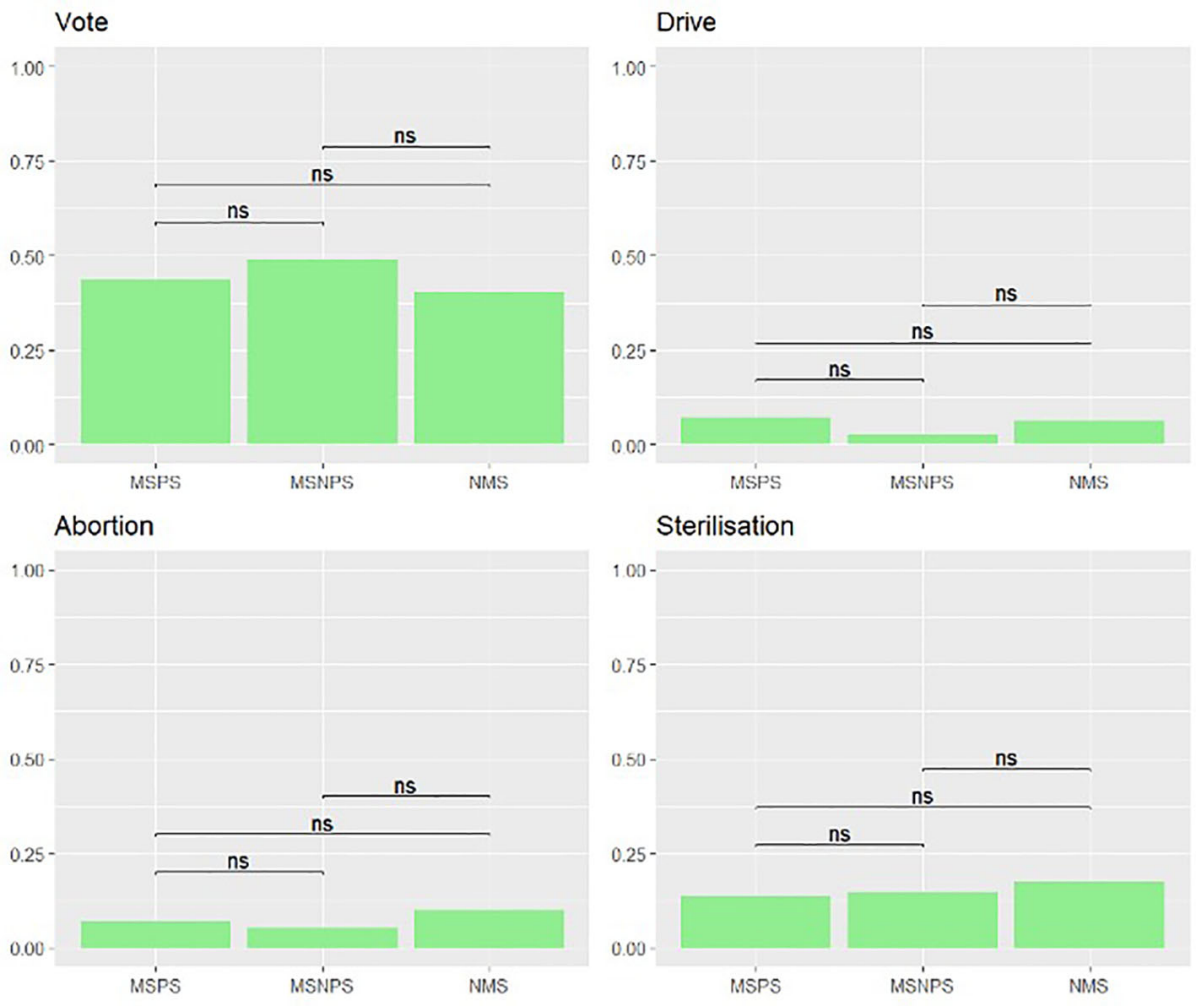
Restrictions questionnaire - Secondary analysis. Secondary analysis for each item of the Restrictions questionnaire with group comparisons with Chi-Square tests with 1 signifying agreement and 0 disagreement with the restriction of a specific right. MSPS, Medical student with psychiatry course; MSNPS, Medical students without psychiatry course; NMS, Non-medical students. ns, non-significant.

Restriction questionnaire responses per group, per item can be found in **Table 2**; both logistic regression models for overall agreement with restrictions can be found in the **Supplementary Material**. There were no significant differences between the attitudes of the three observed student groups (MSNPS vs. MSPS: OR = 0.68, p = 0.08; NMS vs. MSPS: OR = 1.16, p = 0.7; MSNPS vs. NMS: OR = 0.59, p = 0.12) and no socio-demographic covariates significantly influenced these attitudes (see **Supplementary Table 1**). Furthermore, no difference in attitudes was seen in the secondary analysis investigating group differences on the item level, as displayed in **Figure 1**.

**Table 2:** Restrictions questionnaire – per-group responses.

				MSPS vs. MSNPS		MSPS vs. NMS		MSNPS vs. NMS	
Variable	MSPS^a^	MSNPS^a^	NMS^a^	Chi-Square	p-value	Chi-Square	p-value	Chi-Square	p-value
Vote	95 (57%)	114 (51%)	131 (60%)	0.93	0.3	0.29	0.6	3.04	0.081
Drive	12 (7.1%)	6 (2.7%)	14 (6.4%)	3.37	0.066	0.01	>0.9	2.70	0.10
Abortion	12 (7.1%)	12 (5.4%)	22 (10%)	0.26	0.6	0.67	0.4	2.76	0.10
Sterilisation	23 (14%)	33 (15%)	38 (17%)	0.03	0.9	0.70	0.4	0.36	0.5

MSPS, Medical student with psychiatry course; MSNPS, Medical students without psychiatry course; NMS, Non-medical students. For columns 4 to 9, *χ²-*tests were calculated for each possible between-group comparison and the statistics for each test are given.

a n (%) of pro-restriction responses.

Next, the attitudes toward compulsory admissions of psychiatric patients were analyzed; the results per group, per item are displayed in **Table 3** and both logistic regression models for overall agreement with compulsory admissions are displayed in the **Supplementary Material**.

**Table 3:** Compulsory admissions questionnaire – per-group responses.

				MSPS vs. MSNPS		MSPS vs. NMS		MSNPS vs. NMS	
Variable	MSPS^a^	MSNPS^a^	NMS^a^	Chi-Square	p-value	Chi-Square	p-value	Chi-Square	p-value
Suicide	164 (98%)	216 (97%)	177 (81%)	0.02	0.9	24.03	<0.001	27.23	<0.001
Violence	165 (98%)	209 (94%)	186 (85%)	3.63	0.057	18.34	<0.001	8.08	0.004
Care	156 (93%)	196 (88%)	181 (83%)	2.11	0.15	7.92	0.005	2.02	0.2
Delusion	158 (94%)	180 (81%)	182 (83%)	13.41	<0.001	9.67	0.002	0.28	0.6
Disturbance	160 (95%)	200 (90%)	181 (83%)	3.32	0.068	13.21	<0.001	4.03	0.045
Isolation	160 (95%)	188 (84%)	164 (75%)	10.61	0.001	27.42	<0.001	5.48	0.019
Medication	140 (83%)	129 (58%)	134 (61%)	27.82	<0.001	21.50	<0.001	0.38	0.5
Family	86 (51%)	87 (39%)	135 (62%)	5.28	0.022	3.83	0.050	21.74	<0.001

MSPS, Medical student with psychiatry course; MSNPS, Medical students without psychiatry course; NMS, Non-medical students. For columns 4 to 9, *χ²-*tests were calculated for each possible between-group comparison and the statistics for each test are given.

a n (%) of pro-admission responses.

The majority of the study population endorsed compulsory admissions under certain conditions (91.6%). A significant group difference was seen between MSNPS and MSPS (OR = 3.21; p = 0.006), as well as MSNPS and NMS (OR = 3.30; p = 0.033), with MSPS and NMS being significantly less in favor of compulsory admissions than MSNPS. No significant difference was seen between the attitudes of NMS and MSPS towards compulsory admissions. Additionally, socio-demographic covariates in both models did not significantly influence these attitudes (see **Supplementary Table 2**).

**Figure 2:**
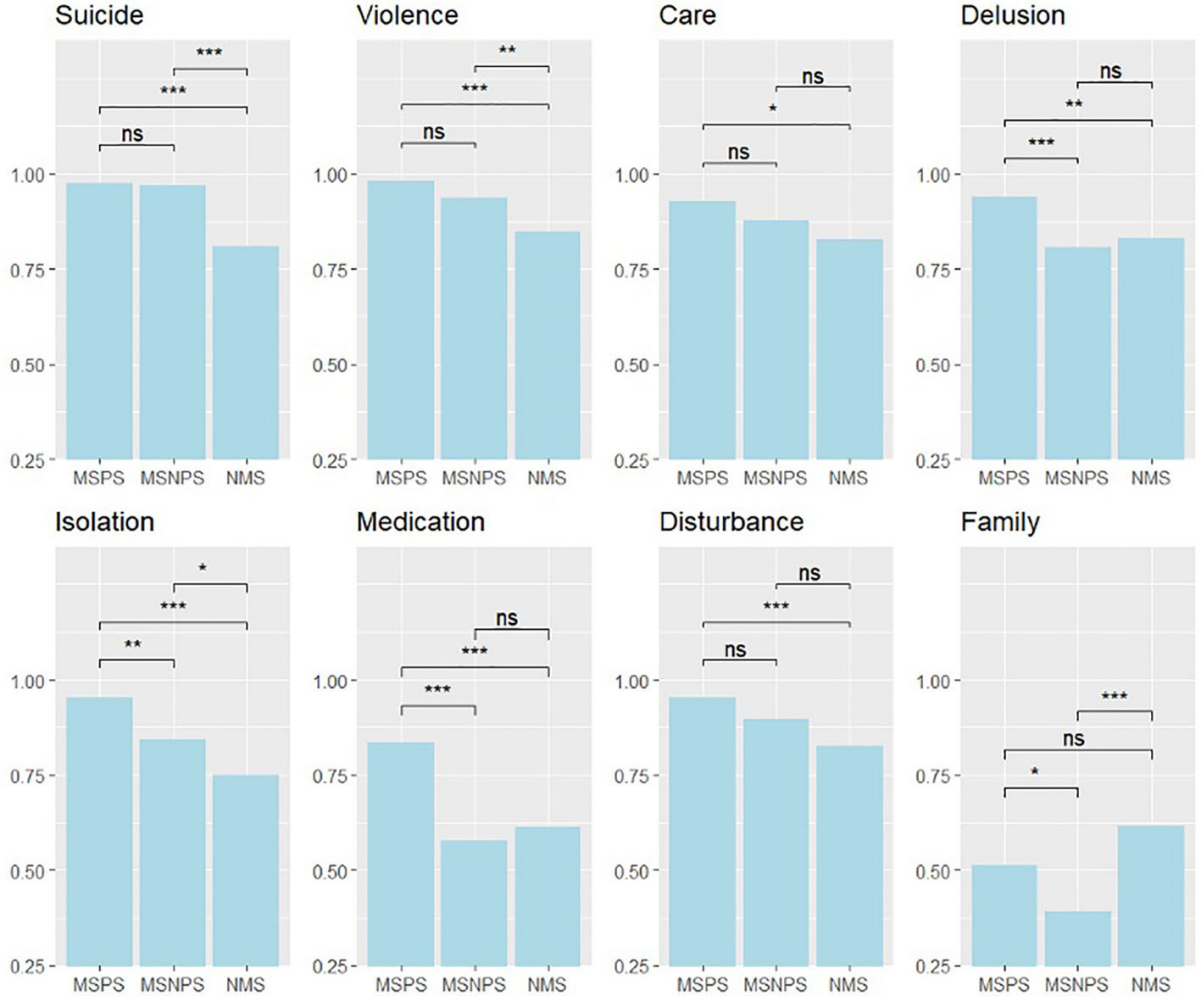
Compulsory Admissions questionnaire – secondary analysis. Secondary analysis for each item of the compulsory admissions questionnaire with group comparisons with Chi-Square tests; MSPS, Medical student with psychiatry course; MSNPS, Medical students without psychiatry course; NMS, Non-medical students. ns, non-significant, * ≤ 0.05, ** ≤ 0.01, *** ≤ 0.001.

The secondary analysis assessing group differences on single items about compulsory admissions showed that, generally, MSPS were more likely to endorse compulsory admissions in specific situations than MSNPS and NMS (**Figure 2**). The differences between attitudes of MSPS and MSNPS were especially relevant for delusions (*χ²* = 13.41; p <.001), social withdrawal (*χ²* = 10.61; p = 0.001), non-adherence to prescribed medication (*χ²* = 27.82; p <.001), and if the family requested compulsory admission (*χ²* = 5.28; p = 0.022). MSPS were significantly more likely to endorse compulsory admissions in all scenarios than NMS except if the family of a mentally ill person wanted it this way. In this situation, NMS were significantly more likely to approve compulsory admission than MSNPS (*χ²* = 21.74; p <.001). MSNPS were more likely to endorse compulsory admissions than NMS if patients were suicidal (*χ²* = 27.23; p <.001), violent towards others (*χ²* = 8.08; p = 0.004), caused a disturbance (*χ²* = 4.03; p = 0.045) or withdrew socially (*χ²* = 5.48; p = 0.019).

## 4 Discussion

This study investigated attitudes of Vietnamese students towards restrictions of rights and compulsory admissions of psychiatric patients. Generally, international studies on these questions are limited, especially in the Global South. The existing comparable studies have been considered here to embed this research’s findings into a global context, albeit being geographically limited to a few European and Vietnamese studies. Overall, Vietnamese students in the present study showed lower acceptance rates of restrictions of mentally ill people than in comparable studies from Switzerland (49) or Germany (22). The same is true when comparing attitudes towards restrictions with a study of the general population in the Hanoi urban area (32). Here, researchers found that 11.9% of respondents were in favor of revoking a driver’s license from a mentally ill person compared to 5.2% of students in the present study. 7.5% of students were in favor of abortion if a mentally ill female becomes pregnant, compared to 21.4% of the general population, and 15.4% of students, compared with 21.6% of the general population, agreed with sterilizing mentally ill persons (32). Previous studies have shown that endorsement of abortion was higher in older population groups, thus providing an explanation for the lower rates in the current study (32). Although numbers are generally lower than in the general population, the approval rate of such invasive procedures like abortion or sterilization is still substantial. This may be due to Vietnamese societal beliefs that the development of the child is strongly influenced by the physical and emotional state of the mother in pregnancy (50) resulting in more restrictive views on childbearing in psychiatric patients.

Furthermore, it is striking to find that more than half of respondents endorsed restricting psychiatric patients’ right to vote, especially when considering that Vietnam adopted the UN Convention on the Rights of People with Disabilities (CRPD) in 2014 (51). The convention affirms that persons with disabilities, including persons with severe mental illness, are to be guaranteed full enjoyment of human rights and fundamental freedoms without discrimination, including the right and opportunity to vote. These attitudes may be explained by the fact that severely mentally ill patients in Vietnam present more exacerbated clinical conditions due to the country’s psychiatric infrastructure (1). Due to limited clinical and outpatient facilities, where psychiatric disorders can be treated earlier, conditions may worsen, leading to a more negative public perception of mental disorders. A recent study on the belief systems of the general population of the Hanoi region about patients with schizophrenia and depression showed that a vast majority of participants correctly conceptualized vignettes of the two mentioned diagnoses as mental disorders. Nevertheless, the majority was not able to correctly identify the diagnoses, nor explicitly recommended medical or psychological treatment (52). This indicates that overall, an understanding of severe mental illness is present in the general population but knowledge on appropriate treatment possibilities is still lacking. In the scope of Vietnam’s National Mental Health Strategy 2015–2025, measures are being implemented to scale up mental health services and psychological interventions (53). The study further underlines the importance of these measures to ensure equal civil rights for psychiatric patients.

Vietnamese students were more accepting of compulsory admissions of psychiatric patients compared to the general population in other national and international studies. For instance, a survey of the change in public opinions of the general German population between the years 1993 and 2011 towards compulsory admissions showed acceptance rates of 74% in 1993 and 71% in 2011 compared to 91.6% in the present study (22). A study in the Swiss population, also showed lower acceptance rates than in the present study, ranging between 68 and 78% (54). Vietnamese students also showed higher acceptance rates of compulsory admissions than the general population in the greater Hanoi urban area (32). This may be due to the difference in academic levels in the two populations, with the study sample solely including university students with a median age of 22, which is in line with previous studies, demonstrating that higher education increased the acceptance of compulsory admission (54–56). Another study conducted in Germany by Zogg et al. underlines this, showing that involuntary admissions were accepted by 98.9% of psychiatrists and only 72.2% of non-specialists (57). Higher education levels may be associated with lower stigma towards mental illnesses and higher levels of trust in the psychiatric care system, thus increasing endorsement of compulsory admissions. Overall, these socio-demographic factors (i.e., higher levels of educational attainment and younger age) have been shown to be associated with a reluctance to accept civil rights infringements of psychiatric patients and be more accepting of involuntary admissions (22, 32, 49, 54, 57).

On the other hand, contrasting to previous studies, an influence of gender on attitudes was not found in the present study (22, 32, 49, 54). This may indicate that gender differences become less important with higher education levels (58). For instance, the increased risk for mental health disorders in women in Vietnam has been shown to be significantly reduced when controlling for years of education (59). Studies on gender roles in Vietnam show that high education levels of women are associated with a more equal distribution of caretaking tasks between genders, and traditional views on female and male roles are more widely spread in the older population (60, 61). The high endorsement of compulsory admissions may also reflect a high level of trust of Vietnamese students in the psychiatric health care system.

No significant differences appeared between the student groups when comparing overall attitudes on restrictions. In contrast, MSPS and MSNPS differed significantly in their attitudes toward compulsory admissions, with MSNPS showing overall significantly stronger agreement with this measure than MSPS and NMS. MSPS and NMS showed comparable attitudes. However, when assessing attitudes on specific scenarios in which psychiatric patients may be involuntarily admitted to in-patient treatment, MSPS were more likely to endorse compulsory admission (in case of suicidal ideation, violence towards others, neglecting self-care, delusions, social withdrawal, non-adherence to prescribed medication, or causing a public disturbance) than MSNPS and NMS. This, however, was not true for the item “A mentally ill person should be admitted against his will if the family wants it this way.” Here, NMS were significantly more likely to agree with compulsory admission than MSNPS and showed higher levels of agreement than MSPS. This may reflect the fact that with increasing mental health literacy, professional medical judgment is more important than families’ opinions when deciding on compulsory admission. This is in line with a previous study on the impact of a psychiatry course on Vietnamese medical students’ attitudes toward psychiatric patients (9). Additionally, witnessing more severely ill patients in clinical rotations may reinforce agreement with involuntary treatment.

Nevertheless, interpretation of associations between attending a psychiatry course on students’ attitudes is limited due to the assessment via self-report questionnaires. This may lead to a social desirability bias as respondents may answer questions in a manner they believe is more acceptable or desirable rather than providing their true thoughts or feelings. This may further be compounded by the fact that the study was conducted by the Department of Psychiatry of the Hanoi Medical University, possibly leading to an over-reporting of positive attitudes. Additionally, the employed questionnaires used theoretically derived items and not psychometrically tested instruments.

Moreover, the current study was limited to the urban area of Hanoi. Future studies should expand the geographical scope to other Vietnamese universities, and specifically, more rural areas of Vietnam. Additionally, a randomized controlled longitudinal study design could shine a light on the causal influence of a psychiatry course on medical students’ attitudes, and qualitative methods could support the interpretation of results. In addition to a longitudinal design, including a follow-up to assess the long-term effect of stigma-reducing measures would be beneficial. A behavioral assessment could help evaluate how the change in attitudes toward psychiatric patients impacts their behavior toward them.

In conclusion, Vietnamese students showed lower levels of acceptance of restrictions and higher levels of acceptance of compulsory admissions rights in psychiatric patients compared to the general population in the Hanoi urban area and international comparisons. Furthermore, we provide tentative evidence that a psychiatry course may encourage more differentiated opinions on the use of compulsory admissions in psychiatric care. Psychoeducation may be a useful tool to reduce mental health stigma. This underlines the importance of introducing courses with direct clinical contact with psychiatric patients into the medical curriculum. The psychiatry courses should be spread more broadly, specifically to other universities, considering they are currently only part of the medical curriculum at Hanoi Medical University. Additionally, education on mental health should be introduced earlier, such as in school, and in a broader sense, such as in the workplace to increase mental health literacy.

Further research, particularly in a longitudinal study design, is needed to explore causal relationships and the long-term effects of a psychiatry course on medical students’ attitudes toward psychiatric patients in Vietnam.

## Data Availability

The raw data supporting the conclusions of this article will be made available by the authors, without undue reservation.
