# Supplementary material for: Attitudes of Vietnamese University students on restrictions of rights and compulsory admissions in patients with severe mental illness – a cross-sectional study
Source: Front Psychiatry. 2025 Mar 19;16:1542247. doi: 10.3389/fpsyt.2025.1542247 (PMC11973515; doi:10.3389/fpsyt.2025.1542247)
Supplement: Supplementary file 1 [file Table1.docx]

## Supplementary Material: Attitudes of Vietnamese University Students on Restrictions of Rights and Compulsory Admissions with Severely Mental Illness – A Cross-Sectional Study

**Table 1.**

*Demographics of study sample*

|  | | | | MSPS vs. MSNPS | | MSPS vs. NMS | | MSNPS vs. NMS | |
| --- | --- | --- | --- | --- | --- | --- | --- | --- | --- |
| Variable | MSPS,  N = 168^a^ | MSNPS,  N = 223^a^ | NMS,  N = 219^a^ | test  statistic^b^ | p-value | test  statistic^b^ | p-value | test  statistic^b^ | p-value |
| Gender |  |  |  | 3.17 | 0.075 | 4.95 | 0.026 | 0.16 | 0.7 |
| Male | 72 (43%) | 117 (52%) | 120 (55%) |  |  |  |  |  |  |
| Female | 96 (57%) | 106 (48%) | 99 (45%) |  |  |  |  |  |  |
| Age | 22.49 (0.66) | 22.09 (0.32) | 19.50 (1.08) | 7.26 | <0.001 | 33.74 | <0.001 | 34.15 | <0.001 |
| Living area |  |  |  | 21.02 | <0.001 | 2.27 | 0.13 | 10.43 | 0.001 |
| < city | 63 (38%) | 137 (61%) | 100 (46%) |  |  |  |  |  |  |
| City | 105 (63%) | 86 (39%) | 119 (54%) |  |  |  |  |  |  |
| Relationship status |  |  |  | 2.92 | 0.087 | 13.33 | <0.001 | 3.97 | 0.046 |
| In partnership | 70 (42%) | 73 (33%) | 52 (24%) |  |  |  |  |  |  |
| No partner | 98 (58%) | 150 (67%) | 167 (76%) |  |  |  |  |  |  |
| Semester |  |  |  |  |  | 231.87 | <0.001 | 280.71 | <0.001 |
| < fourth year | 0 (0%) | 0 (0%) | 171 (78%) |  |  |  |  |  |  |
| At least fourth year | 168 (100%) | 223 (100%) | 48 (22%) |  |  |  |  |  |  |
| Considers oneself mentally ill | 40 (24%) | 28 (13%) | 24 (11%) | 7.68 | 0.006 | 10.46 | 0.001 | 0.14 | 0.7 |
| Close person has used mental health service | 40 (24%) | 66 (30%) | 61 (28%) | 1.34 | 0.2 | 0.61 | 0.4 | 0.09 | 0.8 |

^a^n (%); Mean (SD)

^b^X^2^; t

**Table 2.**

*Restrictions questionnaire – logistic regression models*

|  | Model 1 | | | Model 2 | | |
| --- | --- | --- | --- | --- | --- | --- |
| Variable | OR^1^ | 95% CI^1^ | p-value | OR^1^ | 95% CI^1^ | p-value |
| Group |  |  |  |  |  |  |
| MSPS | — | — |  | 0.86 | 0.42, 1.70 | 0.7 |
| MSNPS | 0.68 | 0.45, 1.05 | 0.082 | 0.59 | 0.29, 1.14 | 0.12 |
| NMS | 1.16 | 0.59, 2.37 | 0.7 | — | — |  |
| Gender |  |  |  |  |  |  |
| Male | — | — |  | — | — |  |
| Female | 0.77 | 0.55, 1.08 | 0.13 | 0.77 | 0.55, 1.08 | 0.13 |
| Relationship status |  |  |  |  |  |  |
| In partnership | — | — |  | — | — |  |
| No partner | 0.76 | 0.52, 1.09 | 0.14 | 0.76 | 0.52, 1.09 | 0.14 |
| Living area |  |  |  |  |  |  |
| < city | — | — |  | — | — |  |
| City | 0.87 | 0.61, 1.22 | 0.4 | 0.87 | 0.61, 1.22 | 0.4 |
| Semester |  |  |  |  |  |  |
| < fourth year | — | — |  | — | — |  |
| At least fourth year | 1.06 | 0.54, 2.18 | 0.9 | 1.06 | 0.54, 2.18 | 0.9 |
| *Note.* OR = Odds Ratio, CI = Confidence Interval, MSPS = Medical student with psychiatry course, MSNPS = Medical students without psychiatry course, NMS = Non-medical students. In model 1, MSPS is used as a reference for the grouping variable; in model 2, NMS is used as a reference. All covariates remain the same for both models.  ^a^OR and CI for a given predictor. | | | | | | |

## Table 3.

*Compulsory admissions questionnaires – logistic regressions models*

|  | Model 1 | | | Model 2 | | |
| --- | --- | --- | --- | --- | --- | --- |
| Variable | OR^1^ | 95% CI^1^ | p-value | OR^1^ | 95% CI^1^ | p-value |
| Group |  |  |  |  |  |  |
| MSPS | — | — |  | 1.03 | 0.36,  2.60 | >0.9 |
| MSNPS | 3.21 | 1.44,  7.70 | 0.006 | 3.30 | 1.04,  9.76 | 0.033 |
| NMS | 0.97 | 0.38,  2.81 | >0.9 | — | — |  |
| Gender |  |  |  |  |  |  |
| Male | — | — |  | — | — |  |
| Female | 1.24 | 0.69,  2.25 | 0.5 | 1.24 | 0.69,  2.25 | 0.5 |
| Relationship status |  |  |  |  |  |  |
| In partnership | — | — |  | — | — |  |
| No partner | 0.65 | 0.32,  1.25 | 0.2 | 0.65 | 0.32,  1.25 | 0.2 |
| Living area |  |  |  |  |  |  |
| < city | — | — |  | — | — |  |
| City | 0.76 | 0.41,  1.38 | 0.4 | 0.76 | 0.41,  1.38 | 0.4 |
| Semester |  |  |  |  |  |  |
| < fourth year | — | — |  | — | — |  |
| At least fourth year | 0.68 | 0.26,  2.00 | 0.4 | 0.68 | 0.26,  2.00 | 0.4 |

*Note.* OR = Odds Ratio, CI = Confidence Interval, MSPS = Medical student with psychiatry course, MSNPS = Medical students without psychiatry course, NMS = Non-medical students. In model 1, MSPS is used as a reference for the grouping variable; in model 2, NMS is used as a reference. All covariates remain the same for both models.

^a^OR and CI for a given predictor.
